# Exploring the effectiveness of artificial intelligence, machine learning and deep learning in trauma triage: A systematic review and meta-analysis

**DOI:** 10.1177/20552076231205736

**Published:** 2023-10-09

**Authors:** Oluwasemilore Adebayo, Zunira Areeba Bhuiyan, Zubair Ahmed

**Affiliations:** 1 Institute of Inflammation and Ageing, 150183 College of Medical and Dental Sciences, University of Birmingham, Edgbaston, Birmingham, UK; 2 Centre for Trauma Sciences Research, 572807 University of Birmingham, Edgbaston, Birmingham, UK

**Keywords:** Artificial intelligence, machine learning, deep learning, trauma, triage

## Abstract

**Background:**

The development of artificial intelligence (AI), machine learning (ML) and deep learning (DL) has advanced rapidly in the medical field, notably in trauma medicine. We aimed to systematically appraise the efficacy of AI, ML and DL models for predicting outcomes in trauma triage compared to conventional triage tools.

**Methods:**

We searched PubMed, MEDLINE, ProQuest, Embase and reference lists for studies published from 1 January 2010 to 9 June 2022. We included studies which analysed the use of AI, ML and DL models for trauma triage in human subjects. Reviews and AI/ML/DL models used for other purposes such as teaching, or diagnosis were excluded. Data was extracted on AI/ML/DL model type, comparison tools, primary outcomes and secondary outcomes. We performed meta-analysis on studies reporting our main outcomes of mortality, hospitalisation and critical care admission.

**Results:**

One hundred and fourteen studies were identified in our search, of which 14 studies were included in the systematic review and 10 were included in the meta-analysis. All studies performed external validation. The best-performing AI/ML/DL models outperformed conventional trauma triage tools for all outcomes in all studies except two. For mortality, the mean area under the receiver operating characteristic (AUROC) score difference between AI/ML/DL models and conventional trauma triage was 0.09, 95% CI (0.02, 0.15), favouring AI/ML/DL models (*p* = 0.008). The mean AUROC score difference for hospitalisation was 0.11, 95% CI (0.10, 0.13), favouring AI/ML/DL models (*p* = 0.0001). For critical care admission, the mean AUROC score difference was 0.09, 95% CI (0.08, 0.10) favouring AI/ML/DL models (*p* = 0.00001).

**Conclusions:**

This review demonstrates that the predictive ability of AI/ML/DL models is significantly better than conventional trauma triage tools for outcomes of mortality, hospitalisation and critical care admission. However, further research and in particular randomised controlled trials are required to evaluate the clinical and economic impacts of using AI/ML/DL models in trauma medicine.

## Introduction

**Figure 1.**
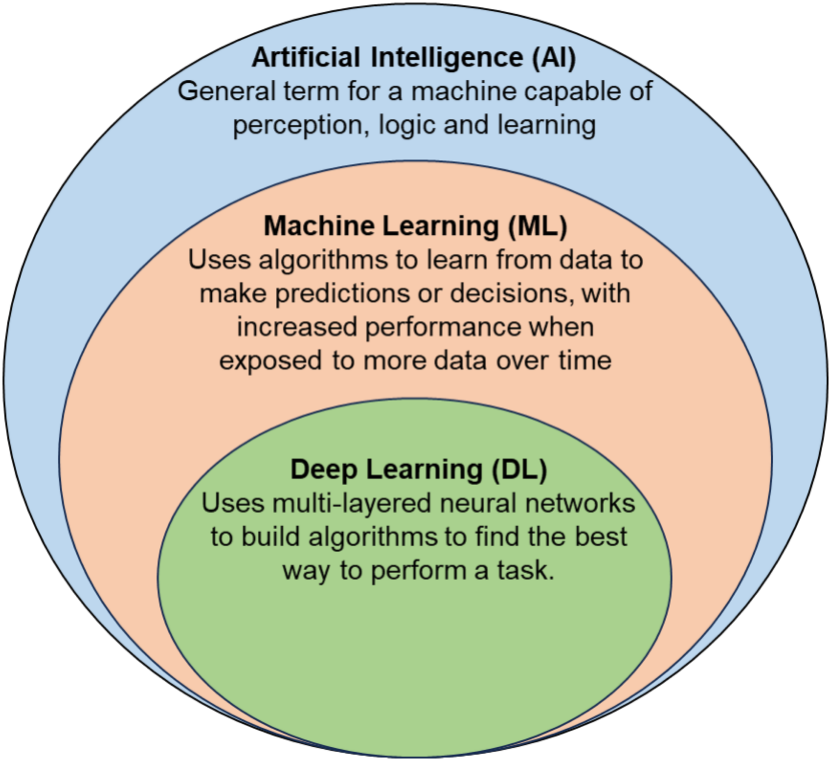
Diagrammatic representation of the relationship between artificial intelligence, machine learning and deep learning. AI: artificial intelligence; ML: machine learning; DL: deep learning.

Technological innovation has been at the forefront of recent global development. Arguably the fastest rate of development has been in the field of artificial intelligence (AI), especially in the medical profession.^1^ AI refers to the capability for inhuman systems to make decisions based on input data (Figure 1).^2^ Machine Learning (ML) is a branch of AI that aims to create decision-making algorithms that gradually improve as they are exposed to data.^2^ The algorithms are then able to recognise vital data motifs for given outcomes which are subsequently stored in model parameters—set values which determine how the model stores and processes data. Deep learning (DL) is a further subset which creates models capable of learning and applying complex data patterns.^2,3^

Most AI models are created using a specific structure, beginning with inputting data from a large database to develop a model with the ability to generate a useful output. This is often used to solve a pre-defined objective. In medicine, these objectives can be patient diagnosis or prognosis,^4^ drug discovery^5^ or note transcription.^6^ AI is commonly employed in visually oriented specialties, such as dermatology, to assist clinicians in detecting skin cancer^4,7^; ophthalmology, for identification and grading of diabetic retinopathy; or radiography, to analyse chest radiographs.^8–10^ However, an underexplored area in which AI may be able to play a major role is in trauma triage.

Triage is the categorisation of patients by healthcare professionals based on the severity of their injuries.^11^ This ensures patients are at the right location with the right resources, at the right time, and are given the correct management.^12,13^ Patients with the greatest risk of preventable adverse outcomes are categorised as Priority 1 (P1); therefore urgent and accurate identification of such patients is vital.^12^ Optimal triage limits preventable disability and death and avoids the overburdening of emergency departments.^14^ Incorrect triage leads to over-triage, when non-critically injured patients are transferred to higher level facilities or under-triage, when critically injured patients are not transferred to a specialised trauma team.^15,16^ Either consequence results in poor patient outcomes, misallocation and overwhelming of emergency and surgical resources.^17^

Currently, conventional triage tools such as the National Early Warning Score, Modified Early Warning Score, Revised Trauma Score (RTS), Trauma and Injury Severity Score (TRISS) and many more are used by physicians depending on hospital guidance.^18,19^ All triage tools require basic physiological data such as respiratory rate, systolic blood pressure, heart rate, capillary refill time and Glasgow Coma Scale.^12,19^ Physicians are then able to merge this knowledge with diagnostic reasoning to determine the patient's potential trauma outcomes and triage destination. This is commonly through the analytical reasoning approach, combining previous knowledge and experience with existing data to make decisions.^20^

However, a limitation of using triage tools is the dependence on the physician's decision making. Whilst this is often accurate, it can be compromised due to the high levels of stress common in trauma care. In addition, certain triage tools require detailed physical examinations or history taking, which may be susceptible to physician variability.^21^ This creates a system where the accuracy of triage tools is dependent on a physician's level of experience and skill.

Utilising the prognostic predictive abilities of AI, ML and DL, combined with the increasing availability of large trauma databases such as the Trauma Audit & Research Network^22^ may offer an avenue to overcome the limitations of conventional triage tools. Whilst there is a review by Liu, 2014 which evaluates ML for predicting outcomes in trauma,^23^ there are no systematic reviews to date which analyse the effectiveness of various AI, ML and DL models in trauma triage. Therefore, this systematic review aimed to critically appraise the effectiveness of AI, ML and DL models at predicting outcomes in trauma triage. A meta-analysis was further performed to assess the accuracy of AI, ML and DL models predicting outcomes of mortality, hospitalisation and critical care admission compared to conventional triage tools.

## Methods

### Search strategy and selection criteria

This systematic review and meta-analysis was performed in accordance with the Preferred Reporting Items for Systematic Reviews and Meta-Analysis (PRISMA) guidelines.^24^ The systematic review was not registered in PROSPERO or any other database.

Two reviewers (OA and ZA) independently searched PubMed, Ovid MEDLINE, ProQuest and Embase databases for primary research published from 1 January 2010 to 9 June 2022. The search was performed on 9 June 2022. A tailored systematic search strategy consisting of Medical Subject Headings (MeSH) including keywords such as ‘triage’, ‘artificial intelligence’, ‘deep learning’ and ‘machine learning’ was created for each database. The full search strategy for all the databases is found in Table 1. OA also examined the bibliographies of relevant articles identified during the initial search for additional studies.

**Table 1.** Search terms for all databases.

Database	Search Strategy	Limits
Ovid MEDLINE	"triage”.ti,ab. (21422) trauma.ti,ab. (250718) exp Artificial Intelligence/ or exp Machine Learning/ or exp Pattern Recognition, Automated/ (159322) 1 and 2 and 3 (36)	No language restrictions Human subjects From 1 January 2010 to June 2022
Embase	exp machine learning/ or exp algorithm/ or exp mathematical model/ (1054819) exp emergency health service/ (118028) “triage”.ti,ab. (33634) trauma.mp. or exp injury/ (2539545) artificial intelligence.mp. or exp artificial intelligence/ (67000) 1 and 2 and 3 and 4 and 5 (22)	No language restrictions Human subjects From 1 January 2010 to June 2022
ProQuest	noft(artificial intelligence) AND noft(trauma) AND noft(triage) AND noft(machine learning) (46)	No language restrictions Human subjects From 1 January 2010 to June 2022
PubMed	(((artificial intelligence) AND (trauma)) AND (triage)) AND (machine learning) (10)	No language restrictions Human subjects From 1 January 2010 to June 2022

The inclusion criteria were studies which evaluated the use of AI, ML or DL models for trauma triage and compared their effectiveness to conventional trauma triage tools or other AI/ML/DL models. Studies which used AI/ML/DL models for other uses except trauma triage or studies which only developed AI/ML/DL models without validation or testing were excluded. This review was limited to human studies regardless of age, gender, ethnicity or primary presenting complaint. Randomised controlled trials, observational studies, cohort studies and case series were included. Studies with animal subjects or presenting duplicate data were excluded. A detailed selection criteria can be found in Table 2.

**Table 2.** Detailed inclusion and exclusion criteria.

	Inclusion criteria	Exclusion criteria
Population	Human regardless of age, gender, ethnicity, presenting complaint.	Animal studies
Intervention	The use of artificial intelligence, machine learning or deep learning models in the clinical environment for trauma triage	Artificial intelligence used for another purpose other than trauma triage e.g. … surgical planning. Studies which only develop AI/ML/DL models without any validation or testing
Comparison	Triage tools commonly used in trauma scenarios Other artificial intelligence or machine learning models	Studies comparing triage tools but no artificial intelligence
Outcome	Outcome measures including in-hospital mortality (Primary outcome), critical care admission, requirement for in-patient hospitalisation, Patients correctly identified as priority 1, prediction of injury severity, prediction of shock	All outcome measures must be relatable to the use of artificial intelligence in the context of triage
Study type	Randomised control trials Cohort studies Case series Observational studies	Case reports, abstracts, literature reviews, systematic reviews.

After removal of duplicate studies; title and abstract screening of remaining studies was performed by OA and ZAB based on the selection criteria, followed by full-text screening. Any disagreements over study selection were resolved through discussion with ZA

### Data analysis

The following data was then extracted for all included studies using a data extraction spreadsheet: study design, location, study population, study size, AI/ML/DL model, primary outcomes and secondary outcomes and the comparison trauma triage tool(s) or AI/ML/DL model(s).

The primary outcome for this review was prediction of in-hospital mortality. The main secondary outcomes were the prediction of critical care admission and in-patient hospitalisation. The primary effect measure collected for all outcomes was the area under the receiver operating characteristic (AUROC). It was chosen as it is a quantitative value typically used to evaluate the predictive performance of algorithms.^25^

A risk of bias assessment was conducted using the Risk of Bias in Non-randomised Studies of Interventions (ROBINS-I) tool.^26^ Three reviewers (OA, ZA, ZAB) independently gave a risk of bias score (Low, Moderate, Serious, Critical) for each of the tool's seven domains for all included studies. The overall risk of bias score for a study was based on the highest score received in any of the seven domains. Any disagreement was resolved through discussion with the senior author (ZA).

Assessment of heterogeneity was conducted by examining the differences across studies for methodological heterogeneity. We used Review Manager (RevMan 5.3, Cochrane Informatics & Technology, London, UK) to determine the *Q* and *I*^2^ statistics (in percentage) to establish variation between the studies. A meta-analysis of a subgroup of studies that reported overall mortality rates, hospitalisation and critical care admission requirement using AI/ML/DL, and the best standard tool was conducted in RevMan 5.3 (Cochrane Informatics & Technology), using the dichotomous data function employing a random effects model. Corresponding *p*-values were calculated using a chi-squared test in RevMan 5.3.

### Role of the funding source

There was no funding source for this study.

## Results

**Figure 2.**
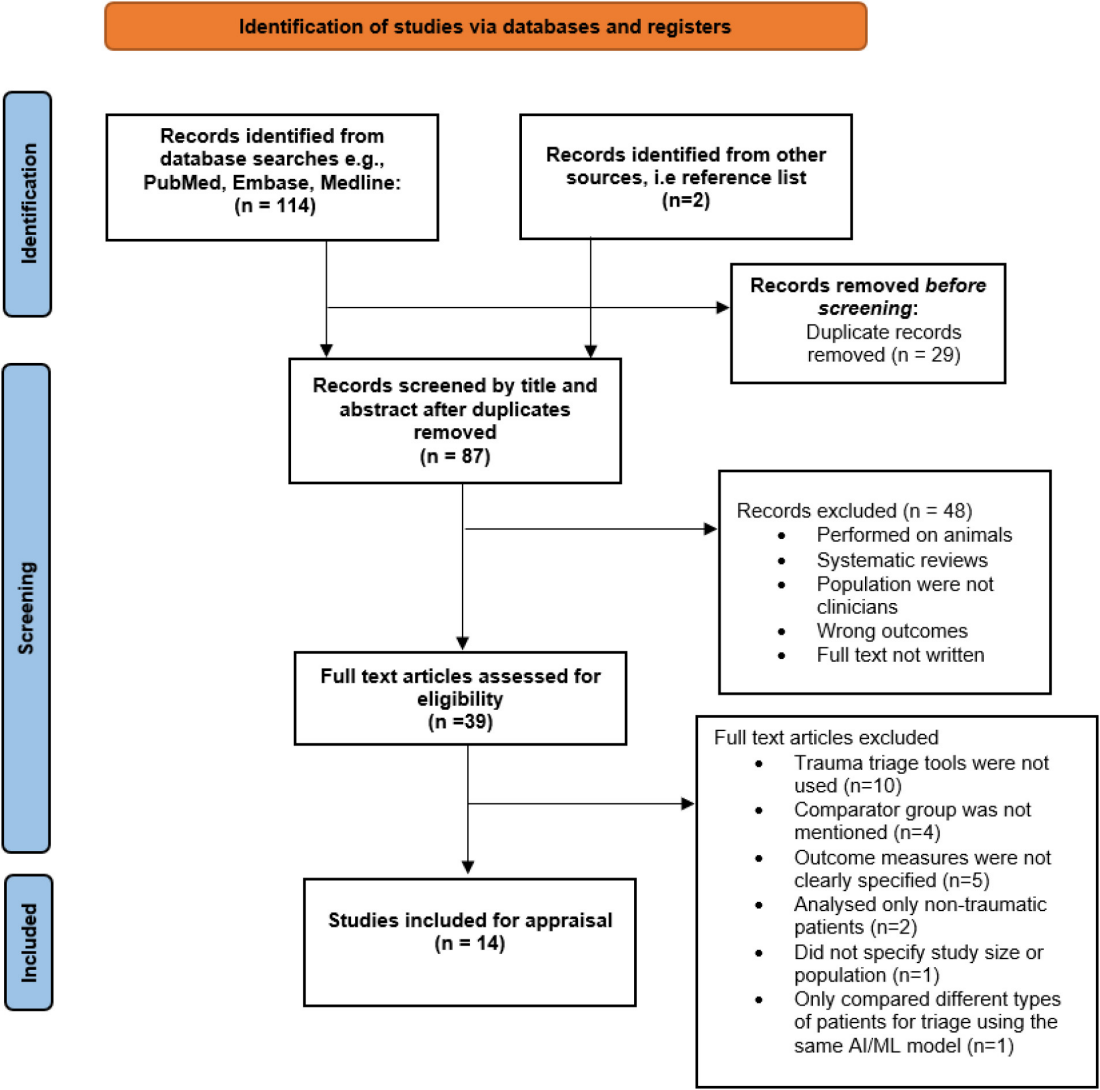
PRISMA study selection flow chart.

The initial search from all databases yielded 114 results, with two additional studies identified from other sources. After removal of duplicates, 87 studies underwent title and abstract screening, with 39 subsequent studies undergoing full-text screening. After full-text screening, a further 25 studies were excluded, resulting in 14 studies being included in this review for analysis.^27–41^ The PRISMA diagram including reasons for exclusion is shown in Figure 2.

All included studies were retrospective observational cohort studies, published between August 2014 and July 2022. The studies all compared specific AI/ML/DL models to either current triage tools, other AI/ML/DL models or a combination of both. The total study size across all studies for both the development and validation of the AI/ML/DL models and trauma triage tools was 29,966,339 patients. The population in all studies was trauma patients who were admitted to the emergency department. Three out of 14 studies utilised data exclusively from paediatric patients (<18 years old),^27,32,36^ whilst the other studies utilised data from only adult trauma patients. An overview of the study characteristics is included in Table 3.

**Table 3.** Overview of study characteristics.

Study	Study design	Location	Population	Study size	Machine Learning /Artificial Intelligence /Deep Learning	Comparison tools	
Mayampurath et al. (2022)^27^	Observational cohort study	USA	ED trauma & non-trauma patients <18 years	Machine Learning model derivation cohort (2009–2017): 1993 patients Machine Learning model validation cohort (2018-2019): 2317 patients	[Machine Learning] Gradient Boosted Machine Learning Model	Bedside Paediatric Early Warning Score	Regularisation Restricted cubic spline regression, Random Forest
Nederpelt et al. (2021)^28^	Retrospective Observational cohort study	USA	Truncal gunshot victims, 16–60yrs	29,816 patients (2015–2017)	[Deep Learning] Information-aware Dirichlet deep neural network (DNN-IAD)/Field artificial intelligence triage (FAIT)	n/a	Logistic regression K-nearest neighbours (kNN) Support vector machines (SVM) Random Forest (RF) Conventional deep neural networks (DNN-CE).
De Hond et al., (2021)^29^	Retrospective Observational cohort study	Netherlands	ED trauma & non-trauma patients > 18 years old	172,104 patients (2017–2019)	[Machine Learning] Gradient Boosted Decision Trees Machine Learning Model (XGBoost) (2017–2019)	n/a	Logistic regression model
Li et al. (2021)^30^	Retrospective Observational cohort study	USA	Blunt & Penetrating trauma patients, 16–89 years	*AI Derivation group:* 1,366,881 patients *AI Validation group:* 449,842 patients (2013–2017)	[Deep Learning] Neural network-based GAPSO (NN-GAPSO) Neural network-based CAPSO (NN-CAPSO) Neural network-based CAPO (NN-CAPO)	Trauma Rating Index in Age, Glasgow Coma Scale, Respiratory rate and Systolic blood pressure (TRIAGES) Score Trauma and Injury Severity Score (TRISS); Revised Trauma Score (RTS); New Trauma Score (NTS); Glasgow Coma Scale, Age, and Arterial Pressure (MGAP) score Glasgow Coma Scale, Age, Systolic Blood Pressure (GAP) score	n/a
Paydar et al. (2021)^31^	Retrospective Observational cohort study	Iran	Trauma patients > 16 years old	1107 patients (2014-2015)	[Machine Learning] Bagging	n/a	Support vector machine (SVM) K-nearest neighbour algorithms(kNN) Adaboost Neural network
Joon-Myoung et al. (2021)^32^	Retrospective Observational cohort study	South Korea	Emergency department trauma & non-trauma patients, < 18 years	2,937,078 patients (2014–2016)	[Deep Learning] Custom deep learning algorithm	Paediatric early warning score Conventional triage and acuity system (CTAS)	Random Forest (RF) Logistic regression (LR)
Klug et al. (2020)^33^	Retrospective Observational cohort study	Israel	ED trauma & non-trauma patients > 18 years old	367,219 patients (2012–2018)	[Machine Learning] Gradient boost (XGBoost)	Shock Index (SI) Modified Shock Index (MSI) Aged Shock Index (ASI)	n/a
Kang et al. (2020)^34^	Retrospective Observational cohort study	South Korea	ED trauma & non-trauma patients > 18 years old	*AI Development:* 8,981,181 patients (2014–2016) *AI Validation/Test:* 2604 (2018-2019)	[Machine Learning] Custom Deep Learning AI Ensemble: Custom Deep Learning AI & ESI/KTAS	Emergency Severity Index (ESI) Korean Triage and Acuity System (KTAS) National Early Warning Score (NEWS) Modified Early Warning Score (MEWS)	n/a
Raita et al. (2019)^35^	Retrospective Observational cohort study	Columbia	ED trauma & non-trauma patients > 18 years old	135,470 patients (2007–2015)	[Artificial Intelligence, Machine Learning, Deep Learning] Lasso regression Random Forest (RF) Gradient boosted decision tree Deep neural network	Emergent Severity Index (ESI)	n/a
Goto et al. (2019)^36^	Retrospective Observational cohort study	Columbia	Emergency department trauma & non-trauma patients, < 18 years	52,037 patients (2007–2015)	[Artificial Intelligence, Machine Learning, Deep Learning] Lasso regression Random Forest (RF) Gradient boosted decision tree Deep neural network	Emergent Severity Index (ESI)	n/a
Spangler et al., (2019)^37^	Retrospective Observational cohort study	Sweden	ED trauma & non-trauma patients > 18 years old	*Development data:* 24,608 patients *Test data:* 13,595 patients 38,203 patients (2016–2018)	[Machine Learning] Gradient boosted model (XGboost) based on ambulance data Gradient boosted model (XGboost) based on dispatch data (e.g.,.. from dispatch nurses)	National Early Warning Score (NEWS)	n/a
Kim et al. (2018)^38^	Retrospective Observational cohort study	USA	Penetrating trauma & Blunt trauma ED patients >18 years old	*AI Training cohort:* 414,779 patients *AI Test/Validation cohort:* 46,086 patients (2007–2013)	[Artificial Intelligence, Machine Learning, Deep Learning] Logistic regression (LR), Random Forest (RF) Deep neural network	Revised Trauma Score (RTS) The trauma and injury severity score (TRISS)	n/a
Joon-Myoung et al. (2018)^39^	Retrospective Observational Cohort Study	South Korea	ED trauma & non-trauma patients > 18 years old	10,967,518 patients (2014–2017)	[Deep Learning] Deep-learning-based Triage and Acuity Score (DTAS)	Modified Early Warning Score (MEWS) Korean Triage and Acuity System (KTAS)	Logistic regression (LR) Random Forest (RF)
Liu et al. (2014)^40^	Retrospective Observational Cohort Study	USA	ED trauma & non-trauma patients > 18 years old	104 patients (2011-2012)	[Machine Learning] Custom Machine Learning model combining multivariate regression modelling & ML-based modelling.	n/a	Standard statistically derived multivariate logistic regression models

Seven studies included in-hospital mortality as an outcome,^30,33,35–39^ six studies analysed hospitalisation as an outcome^29,32,35–37,39^ and five studies examined critical care admission as an outcome.^34–37,39^ Meta-analysis was feasible for all three outcomes of mortality, hospitalisation and critical care admission as more than two studies examined each of these outcomes. All studies performed external validation using a different dataset and used similar numerical outcome measures.^41^ The best-performing AI/ML/DL model was compared to non-AI/ML/DL tools for all studies in the meta-analyses for mortality, hospitalisation and critical care admission.

Due to the range of data available on the various trauma databases used to develop the AI models, the studies were able to analyse multiple outcomes. Other outcomes analysed by the studies include prediction of shock, need for early major haemorrhage control surgery, need for early massive transfusion, prediction of injury severity and prediction of the need for life-saving interventions.^28,31,40^ These outcomes were not eligible for meta-analysis as most were only analysed by one study. The best-performing AI/ML/DL models outperformed their comparator trauma triage tools in all outcomes for all studies analysed except for two studies. The same outcome of mortality was assessed and the same triage tool, and the TRISS was used in both studies in which the trauma triage tool outperformed the AI/ML/DL models.^30,38^ An overview of all study outcomes and results can be found in Table 4.

**Table 4.** Overview of the study outcomes.

Study	Study size	ML/AI/DL models	Comparison tools		Outcomes	Algorithm performance compared to comparison
			Triage tools	ML/AI/DL		*Mean area under the receiver operating characteristic curve (AUROC)*
Mayampurath et al. (2022)^27^	*Machine Learning model derivation cohort* (2009-2017): 1993 patients *Machine Learning model validation cohort* (2018-2019): 2317 patients	[Machine Learning] Gradient Boosted Machine Learning Model (XGBoost)	Bedside Paediatric Early Warning Score	n/a	Direct ward to intensive care unit (ICU)/Critical care transfer	*Derivation cohort:* XGBoost: 0.84 Bedside paediatric early warning score: 0.71 p < 0.001 *Validation cohort:* XGBoost: 0.80 Bedside paediatric early warning score: 0.74 p < 0.001
Nederpelt et al. (2021)^28^	29,816 patients (2015–2017)	[Deep Learning] Information-aware Dirichlet deep neural network (DNN-IAD)/Field artificial intelligence triage (FAIT)	n/a	Logistic regression (LR) Random Forest (RF) K-nearest neighbours (kNN) Support vector machines (SVM) Conventional deep neural networks (DNN-CE)	Prediction of Shock Need for early major haemorrhage control surgery Need for early massive transfusion	*Shock:* FAIT: 0.888; LR: 0.876; RF: 0.893; kNN:0.779; SVM: 0.884; DNN-CE: 0.892 *Need for early major haemorrhage control surgery:* FAIT: 0.863; LR: 0.850; RF: 0.865; kNN: 0.680; SVM: 0.859; DNN-CE: 0.864 *Need for early massive transfusion:* FAIT: 0.819; LR: 0.814; RF: 0.826; kNN: 0.725; SVM: 0.820; DNN-CE: 0.828 *Average AUROC for all 3 outcome measures:* FAIT: 0.856; LR: 0.847; RF: 0.861, kNN: 0.728, SVM: 0.854, DN-CE: 0.861
De Hond et al. (2021)^29^	172,104 patients (2017–2019)	[Machine Learning] Gradient Boosted Decision Trees Machine Learning Model (XGBoost) (2017–2019)	n/a	Logistic regression AI model (LR)	Predicting hospitalisation	XGBoost AUROC: 0.84 (0.77–0.88) LR AUROC: 0.82 (0.78–0.86)
Li et al. (2021)^30^	*AI Derivation group:* 1,366,881 patients *AI Validation and test groups:* 449,842 patients (2013–2017)	[Deep Learning] Neural network-based GAPSO (NN-GAPSO) Neural network-based CAPSO (NN-CAPSO) Neural network-based CAPO (NN-CAPO)	Trauma Rating Index in Age, Glasgow Coma Scale, Respiratory rate and Systolic blood pressure (TRIAGES) Score Trauma and Injury Severity Score (TRISS); Revised Trauma Score (RTS); New Trauma Score (NTS); Glasgow Coma Scale, Age, and Arterial Pressure (MGAP) score Glasgow Coma Scale, Age, Systolic Blood Pressure (GAP) score	n/a	Predicting Prehospital mortality	NN-GAPSO: 0.921 (0.918–0.923) NN-CAPSO: 0.911 (0.909–0.913) NN-CAPO: 0.904 (0.902–0.906) RTS: 0.851 (0.848–0.854) NTS: 0.888 (0.885–0.891) MGAP: 0.898 (0.896–0.901) GAP: 0.897 (0.894–0.899) TRIAGES: 0.903 (0.900–0.905) TRISS: 0.934 (0.932–0.936)
Paydar et al. (2021)^31^	1107 patients (2014–2015)	[Machine Learning] Bagging	n/a	Support vector machine (SVM) K-nearest neighbour algorithms(kNN) Adaboost Neural network	Prediction of likely severity of injuries- in first 24hrs	Bagging: 0.9967 ± 0.00 SVM: 0.9924 ± 0.02 KNN: 0.6384 ± 0.02 Adaboost: 0.7581 ± 0.01 Neural network: 0.5160 ± 0.07
Joon-Myoung et al. (2021)^32^	2,937,078 patients (2014–2016)	[Deep Learning] Custom deep learning algorithm	Paediatric early warning score Conventional triage and acuity system (CTAS)	Random Forest (RF) Logistic regression (LR)	Prediction of intensive care unit (ICU)/Critical care admission Predicting hospitalisation	Deep learning algorithm: 0.908 (95% CI, 0.903–0.910) Paediatric early warning score (0.812 [0.803–0.819]), Conventional triage and acuity system: 0.782 (0.773–0.790), Random Forest: 0.881 (0.874–0.890), Logistic regression: 0.851 (0.844–0.858)
Klug et al. (2020)^33^	367,219 patients (2012–2018)	[Machine Learning] Gradient boost (XGBoost)	Shock Index (SI) Modified Shock Index (MSI) Aged Shock Index (ASI)	n/a	Predicting Early mortality- 2 days from ED presentation Short-term mortality- mortality 2–30 days from registration to ED)	*Early mortality* XGboost: 0.962 (95%CI, 0.956–0.968); SI: 0.742 (95%CI, 0.721–0.765); MSI: 0.751 (95%CI, 0.730–0.772); ASI: 0.858 (95%CI, 0.841–0.874) *Short-term mortality:* XGboost: 0.923 (95%CI, 0.919–0.926); SI: 0.664 (95%CI, 0.654–0.675);); MSI: 0.686 (95%CI, 0.675–0.697); ASI: 0.834 (95%CI, 0.827–0.841)
Kang et al. (2020)^34^	*AI Development:* 8,981,181 patients (2014–2016) *AI Validation/Test:* 2604 (2018-2019)	[Machine Learning] Custom Deep Learning AI Ensemble: Custom Deep Learning AI & ESI/KTAS	Emergency Severity Index (ESI) Korean Triage and Acuity System (KTAS) National Early Warning Score (NEWS) Modified Early Warning Score (MEWS)	n/a	Prediction of intensive care unit (ICU)/Critical care admission	AI + ESI: 0.923 (95% CI, 0.920–0.926, p < 0.001) AI + KTAS: 0.909 (95% CI, 0.906–0.912) AI only: 0.867 (95% CI, 0.864–0.871) ESI: 0.839 (95% CI, 0.831–0.846); KTAS: 0.824 (95% CI, 0.815–0.832); NEWS: 0.741 (95% CI, 0.734–0.748); MEWS: 0.696 (95% CI, 0.691–0.699)
Raita et al. (2019)^35^	135,470 patients (2007–2015)	[Artificial Intelligence, Machine Learning, Deep Learning] Lasso regression Random Forest (RF) Gradient boosted decision tree Deep neural network	Emergent Severity Index (ESI)	n/a	Prediction of intensive care unit (ICU)/Critical care admission *(including In-hospital mortality)* Prediction of Hospitalisation *(admission to inpatient care site or direct transfer to an acute care hospital)*	*Critical care:* ESI: 0.74 (95% CI 0.72–0.75); Lasso regression: 0.84 (95% CI, 0.83–0.85); Random Forest (RF): 0.85 ((95% CI, 0.84–0.87); Gradient boosted decision tree: 0.85 (95% CI, 0.83–0.86); Deep neural network: 0.86 (95% CI, 0.85–0.87) *Hospitalisation:* ESI: 0.69 (95% CI, 0.68–0.69); Lasso regression: 0.81 (0.80–0.81); Random Forest (RF): 0.81 (0.81–0.82); Gradient boosted decision tree: 0.82 (95% CI, 0.82–0.83); Deep neural network: 0.82 (0.82–0.83) *All p < 0.001*
Goto et al. (2019)^36^	52 037 patients (2007–2015)	[Artificial Intelligence, Machine Learning, Deep Learning] Lasso regression Random Forest (RF) Gradient boosted decision tree Deep neural network	Emergent Severity Index (ESI)	n/a	Prediction of intensive care unit (ICU)/Critical care admission *(including In-hospital mortality)* Hospitalisation *(admission to inpatient care site or direct transfer to an acute care hospital)*	*Critical care:* ESI: 0.78 (95% CI 0.71–0.85); Lasso regression: 0.84 (0.77–0.91) p < 0.29; Random Forest (RF): 0.85 (0.79–0.91) p < 0.07; Gradient boosted decision tree: 0.84 (0.79–0.92) p < 0.08; Deep neural network: 0.85 (0.78–0.92) p < 0.16 *Hospitalisation:* ESI: 0.73 (0.71–0.75); Lasso regression: 0.78 (0.76–0.80) p < 0.001; Random Forest (RF): 0.80 (0.78–0.81) p < 0.001; Gradient boosted decision tree: 0.80 (0.78–0.81) p < 0.001; Deep neural network: 0.80 (0.78–0.81) p < 0.001
Spangler et al., (2019)^37^	*Development data:* 24608 patients *Test data:* 13595 patients 38203 patients (2016–2018)	[Machine Learning] Gradient boosted model (XGboost) based on ambulance data Gradient boosted model (XGboost) based on dispatch data (e.g.,.. from dispatch nurses)	National Early Warning Score (NEWS)		Prediction of Hospitalisation Prediction of intensive care unit (ICU)/Critical care admission Patient mortality within 2 days	*Hospital admission:* XGboost based on ambulance data: 0.79 (95% CI 0.78-0.79); XGboost based on dispatch data: 0.72 (0.72-0.73); NEWS Score: 0.67 (0.67-0.68) *Critical care:* XGboost based on ambulance data: 0.79 (0.78–0.80); XGboost based on dispatch data: 0.70 (0.68–0.71); NEWS Score: 0.76 (0.75–0.78) *Two-day mortality:* XGboost based on ambulance data: 0.89 (0.87–0.91); XGboost based on dispatch data: 0.79 (0.77–0.81); NEWS Score: 0.85 (0.83–0.87)
Kim et al. (2018)^38^	*AI Training cohort:* 414,779 patients *AI Test/Validation cohort:* 46,086 patients (2007–2013)	[Artificial Intelligence, Machine Learning, Deep Learning] Logistic regression (LR), Random Forest (RF) Deep neural network	Revised Trauma Score (RTS) The trauma and injury severity score (TRISS)	n/a	Prediction of in-hospital mortality	RTS: 0.78 (95% CI 0.775–0.785); LR: 0.88 (0.872–0.880); RF: 0.87 (0.862–0.872); Neural Network: 0.89 (0.882–0.890); TRISS 0.90 (0.901–0.909) *All p < 0.001*
Joon-Myoung et al. (2018)^39^	10,967,518 patients (2014–2017)	[Deep Learning] Deep-learning-based Triage and Acuity Score (DTAS)	Modified Early Warning Score (MEWS) Korean Triage and Acuity System (KTAS)	Logistic regression (LR) Random Forest (RF)	Prediction of In-hospital mortality Prediction of intensive care unit (ICU)/Critical care admission Prediction of Hospitalisation	*In-hospital mortality:* DTAS: 0.935 (95% CI 0.935-0.936); KTAS: 0.785 (0.785-0.786); MEWS: 0.810 (0.809-0.810); RF: 0.910 (0.910-0.910); LR: 0.903 (0.902-0.903) *Predicting critical care:* DTAS: 0.894 (95% CI 0.894 -0.895); KTAS: 0.797 (0.797-0.797); MEWS: 0.726 (0.725-0.726); RF: 0.822 (0.821 -0.822); LR: 0.818 (0.818 -0.818) *Predicting hospitalisation:* DTAS: 0.804 (95% CI 0.803 -0.804); KTAS: 0.681 (0.681-0.681); MEWS: 0.614 (0.614-0.614); RF: 0.738 (0.738 -0.738); LR: 0.713 (0.713-0.713)
Liu et al. (2014)^40^	104 patients (2011-2012)	[Machine Learning] 1. Custom Machine Learning model combining multivariate regression modelling & ML-based modelling	n/a	Standard statistically derived multivariate logistic regression models	Prediction of need for life-saving interventions	*Custom ML Model:* 0.94 *Standard multivariate logistic regression model:* 0.92 p < 0.001

**Figure 3.**
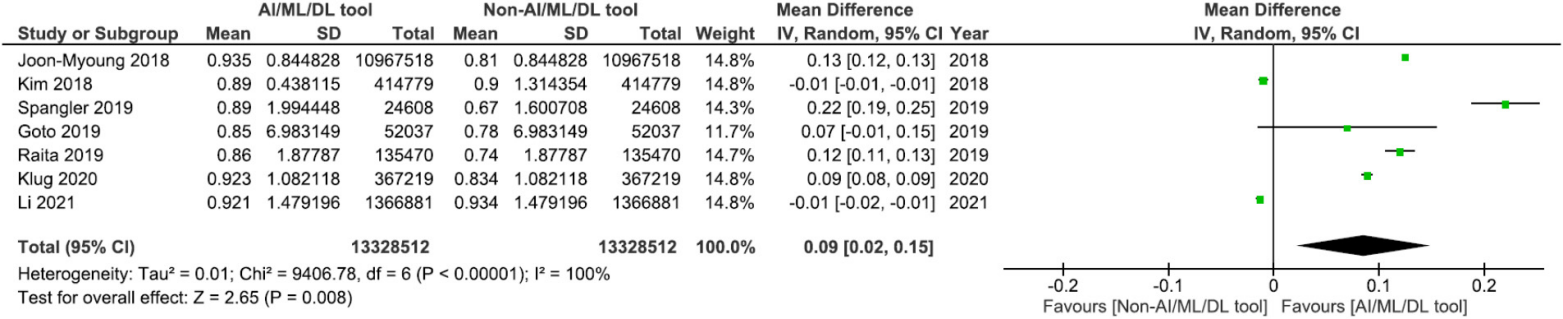
Meta-analysis comparing mortality prediction with AI/ML/DL and non-AI/ML/DL tools. AI: artificial intelligence; ML: machine learning; DL: deep learning.

Five of the seven studies assessing our primary outcome of mortality reported greater AUROC scores in the best-performing AI/ML/DL model compared to the best-performing conventional trauma triage tools.^33,35–37,39^ Four of those five studies reported statistically significantly greater AUROC scores in the AI/ML/DL group compared to the non-AI/ML/DL triage group (*p* < 0.005) (Figure 3).^33,35,37,39^ The mean AUROC score of AI/ML/DL models for mortality was 0.895, whilst the mean AUROC score for the conventional triage tools group was 0.810. Overall, from the meta-analysis, the mean AUROC score difference between the AI/ML/DL models and conventional triage tools was 0.09, 95% CI (0.02, 0.15), in favour of the AI/ML/DL group, with *p* = 0.008 (Figure 3). This suggests that AI/ML/DL models are statistically significantly better at predicting mortality compared to conventional triage tools.

**Figure 4.**
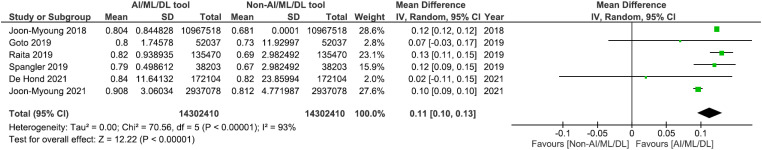
Meta-analysis comparing hospitalisation prediction with AI/ML/DL and non-AI/ML/DL tools. AI: artificial intelligence; ML: machine learning; DL: deep learning.

For the secondary outcome of hospitalisation, all six studies reported greater AUROC scores in the best-performing AI/ML/DL model group compared to the conventional trauma triage group.^29,32,35–37,39^ The AUROC scores in the AI/ML/DL group compared to the non-AI/ML/DL group was statistically significant for four of the six studies (*p* < 0.005) (Figure 4).^29,34,35,39^ The two studies which did not show statistical significance contributed a lower weighting to the meta-analysis due to the imprecision (wider confidence intervals) of their results (Figure 4). The mean AUROC score for the AI/ML/DL group (0.827) was greater than the mean AUROC score for the conventional triage tools group (0.733). Overall, the mean AUROC score difference between the two groups was 0.11, 95% CI (0.10, 0.13) in favour of the AI/ML/DL group, with *p* = 0.00001 (Figure 4). This suggests that AI/ML/DL models are statistically significantly better at predicting hospitalisation compared to conventional triage tools.

**Figure 5.**
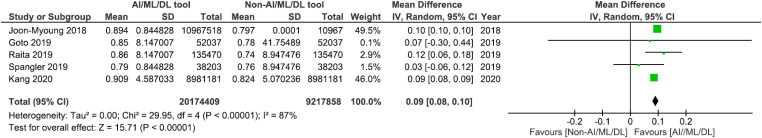
Meta-analysis comparing critical care admission prediction with AI/ML/DL and non-AI/ML/DL tools. AI: artificial intelligence; ML: machine learning; DL: deep learning.

For the other secondary outcome of critical care admission, all five studies reported greater AUROC scores in the best-performing AI/ML/DL model group compared to the conventional trauma triage group.^34–37,39^ Three of the five studies reported significantly greater AUROC scores in the AI/ML/DL group compared to the conventional trauma triage tools group (*p* < 0.005).^34,36,39^ The mean AUROC score of the AI/ML/DL group for critical care admission (0.861) was greater than the mean score for the conventional triage tools group (0.780). Studies by Goto et al., Raita et al. and Spangler et al. contributed a lower weighting to the overall meta-analysis due to result imprecision.^35–37^ The overall mean AUROC score difference between the AL/ML/DL group and the conventional triage tools group was 0.09, 95% CI (0.08, 0.10), favouring the AI/ML/DL group with *p* = 0.00001 (Figure 5). This suggests that AI/ML/DL models are statistically significantly better at predicting critical care admission compared to conventional trauma triage tools.

**Figure 6.**
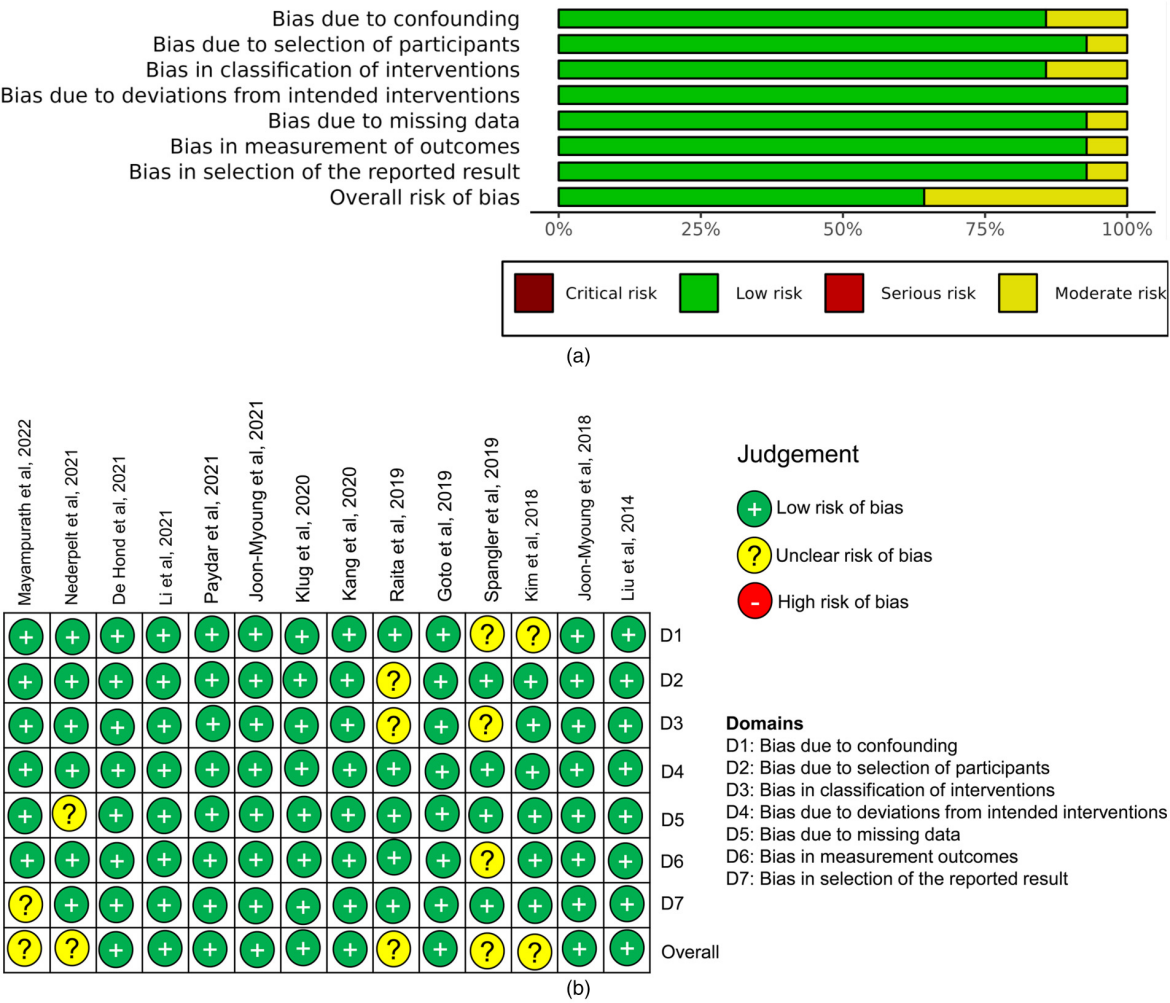
Risk of bias assessment. (a) Summary diagram to show % of articles with bias over the seven domains. (b) Risk of bias in individual studies depicted using the ROBINS-I traffic light plot.

Risk of bias assessment was performed for all 14 studies across the seven domains using the ROBINS-I tool.^26^ Overall, 65% studies were judged as having a moderate risk of bias (Figure 6A). All domains except bias due to deviations from the intended interventions had some studies with a moderate risk of bias. Individually, nine studies had a low risk of bias as bias was accounted for through the use of appropriate regression and standardisation (Figure 6B). In particular, the risk of selection bias was counteracted in these studies by comparing patient characteristics and actual outcomes in the derivation/development (non-analytic) cohort and the external validation (analytic) cohort. Five studies were found to have a moderate risk of bias, commonly misclassification bias due to incorrect data imputation/coding errors or confounding bias as a result of inappropriate/lack of regression.

High heterogeneity, due to varying ages, different populations and different AI/ML/DL models in the meta-analyses of all three outcomes was accounted for using random effects models which counteracted both intra-study and inter-study variance.^42^ This increased the weighting distribution more evenly compared to using the fixed-effects model.

## Discussion

This systematic review and meta-analysis evaluated the ability of AI/ML/DL models to accurately predict trauma outcomes, specifically mortality, hospitalisation and critical care admission. Our results demonstrate that AI/ML/DL models display a better predictive ability for trauma outcomes, particularly mortality, hospitalisation and critical care admission compared to conventional trauma triage tools. Our comprehensive meta-analysis revealed that the difference in predictive ability was statistically significant for all of our outcomes of mortality, hospitalisation and critical care admission. To our knowledge, this is the first systematic review and meta-analysis appraising AI/ML/DL models in comparison to conventional triage tools in the context of mortality, hospitalisation and critical care admission outcomes. These results, therefore, offer a great foundation for the adoption and regular use of AI/ML/DL models in clinical trauma environments.

The overall mean AUROC score differences for the chosen outcomes of mortality, hospitalisation and critical care admission significantly favoured the AI/ML/DL groups compared to the conventional triage tools groups; however, it is important to recognise the difference from the null value was objectively minimal. This suggests the difference between the use of AI/ML/DL for trauma triage at predicting these outcomes is currently statistically significant; however, it is objectively only slightly better compared to the current triage tools. Our meta-analysis for all three outcomes shows that current AI/ML/DL technologies for trauma triage are most effective at predicting hospitalisations as this outcome had the greatest mean AUROC score difference. It can be argued that the ability to predict the other outcomes of critical care admission and mortality have a greater effect on a patient's prognosis.

The results of the meta-analysis for our chosen outcomes signify the potential of a future which has an increased reliance on these AI/ML/DL technologies at predicting mortality, hospitalisation and critical care admission in trauma patients. However, the use of AI/ML/DL for trauma triage is still considered to be in its infancy compared to other well-established methods such as RTS or TRISS.^9,10^ Given there is still a high probability for improvement of these technologies given the speed of recent advancements in AI, it can be surmised the ability of AI/ML/DL models to predict these outcomes with greater accuracy will vastly improve in the future.

A positive finding of this review was the clear improvement in implementation and utilisation of trauma databases globally.^43^ This has been expediated by advancements in health policies, particularly in developing countries, with the establishment of simple, low-cost, electronic trauma databases such as the Nigerian Trauma Registry.^44^ Trauma databases are already well proven to provide vital data which can help guide resource allocation, influence injury prevention approaches and monitor changes in an hospital's trauma system performance.^45^ This combined with the ability of AI/ML/DL models to process and understand large quantities of data rapidly suggests it is feasible to develop, validate and test AI/ML/DL models tailored to different healthcare systems on a large scale. Before this can become fully widespread, implementation of trauma databases both in developed and developing countries must increase. This requires the promotion of a well-defined population, appropriately trained physicians, a reliable data-collection system and the capacity to analyse, report and validate this data.^46^ To accomplish these measures; adequate funding, updated healthcare policies and appropriate resources would be needed, often from government healthcare authorities. Therefore, a future hindrance to the development and implementation of clinical AI/ML/DL models may be a lack of trauma databases.

It is important to highlight that the effectiveness of AI/ML/DL model development is dependent on the choice and type of data acquired from trauma databases.^47^ This was particularly evident in the study by Spangler et al. which found that the AI/ML/DL model developed using ambulance data (patient information acquired from the ambulance team) performed better in all outcomes of hospitalisation, mortality and critical care admission compared to the AI/ML/DL model developed using dispatch data (patient information acquired from the original emergency call) (see Table 2).^37^ This highlights the importance of having trauma databases with high-quality data as this translates to higher quality AI/ML/DL models. This is vital to account for in customised clinical AI/ML/DL models such as in the study by Nederpelt et al., as the regulation in development may be less stringent.^28^ Therefore, it is vital to ensure only the highest quality data is used in model development.

Future development of the best AI/ML/DL systems may require an amalgamation of high-quality conventional triage tools and AI/ML/DL models. This was evident in the study by Kang et al. which assessed a custom DL model, conventional triage tools and a specialised combination of both the custom DL model and conventional triage tools (Ensemble) for the outcome of critical care admission.^34^ It was discovered that whilst the custom DL model outperformed the conventional triage tools, the Ensemble models outperformed both the conventional triage tools and the custom DL model in terms of predictive ability for the study's outcome (see Table 2). Utilisation of this notion may be highly effective when AI/ML/DL are combined with conventional triage tools which appear to offer a high predictive ability such as the TRISS triage tool, the only triage tool from all studies which outperformed the AI/ML/DL models.^30,38^ This introduces the notion that the future of trauma triage may lie not just in the utilisation of AI/ML/DL models but creating methods to integrate the computing power found in these models and the principles of the best-performing conventional triage tools.

This systematic review and meta-analysis has shown that the use of AI/ML/DL models for trauma triage reduces the complexity associated with conventional trauma triage tools which require detailed history taking, physical examinations (e.g. pain score) and physician judgement based on clinical experiences.^21,48^ Most AI/ML/DL models only require imputation of patient variables such as age, sex, primary complaint, trauma type, comorbidities or mental status to determine potential outcomes. This informs patient triage and ideally leads to better patient outcomes.

Another advantage is the fact that the input variables are basic information which can be quickly collected and therefore do not require clinician judgement as this is all processed by the AI/ML/DL models. This would offer clinicians more time to direct towards performing uniquely human skills such as empathy, communication and broad-view problem solving. This also relieves trauma clinicians of time-consuming duties in a speciality in which many physicians are often over-worked and face burnout.^49,50^ This would ultimately lead to improved patient outcomes as clinicians would have more time to perform urgent clinical duties and manage patients to the best of their ability.

It is important to note that whilst this systematic review suggests that AI/ML/DL models can predict trauma patient outcomes with greater accuracy compared to current conventional triage tools, it may not yet be met with confidence from clinicians. This can be due to a lack of education on how AI/ML/DL algorithms work. Therefore, educating trauma physicians on the capabilities and the impact AI/ML/DL models can have and would be an important future step to promote widespread implementation of these models.

A vital consideration for future medical AI/ML/DL models is ensuring that they are transferrable to different hospitals or clinical scenarios. However, this presents a conundrum, similar to the “No Free Lunch” theory for optimisation from Wolpert^51^ which suggests that if an algorithm is optimised for one situation, it may be difficult for it to produce good results in another situation. When applied to our study, it can be inferred if AI/ML/DL models are developed using a particular dataset in a certain environment; it may limit the transferability of that model to a different environment. A way to account for this is through the implementation of internal and external validation in AI/ML/DL algorithms.

All studies in this systematic review were discovered to have undergone validation, with most studies undergoing external validation, using an independent database. In the context of this systematic review, validation should occur after AI/ML/DL model development and can be repeated multiple times with various databases to improve model performance before testing.^23^ A limitation discovered by our systematic review was that there is inconsistency regarding the terminology of validation in various studies. In some studies, the term was used to describe the testing of the final AI/ML/DL models whilst other studies referred to validation as external tuning of the AI/ML/DL models using either an internal or external database.

For future models and studies, we recommend that data cohorts should be clearly distinguished into a development set (to train and develop the AI/ML/DL model), an external validation set (to fine-tune the model using an independent database) and a test set (to assess the performance of the AI/ML/DL model). For external validation to be feasible, data sharing between trauma databases and AI/ML/DL models must be encouraged especially due to the speed at which information evolves on a global scale. However, actions should be taken to ensure the de-identification and anonymisation of patient data in all instances.

**Figure 7.**
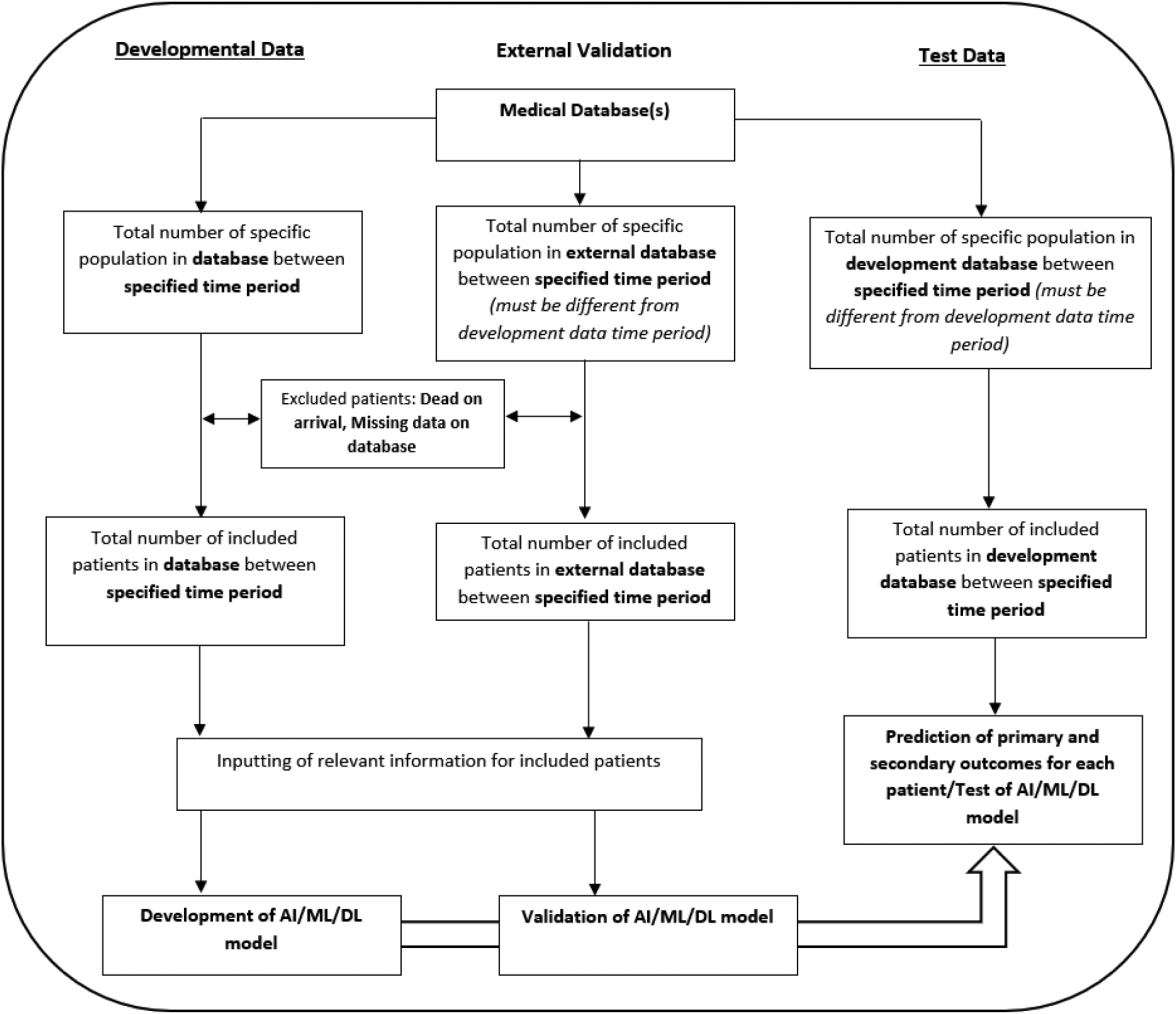
AI/ML/DL algorithm template. AI: artificial intelligence; ML: machine learning; DL: deep learning.

Data validation, ideally external, is important as it ensures AI/ML/DL models are able to showcase the same predictive abilities in diverse populations. This could then lead to the creation of specific trauma triage AI/ML/DL models which can be applied to various populations, similar to AI models used in dermatology to assist clinicians in skin cancer diagnosis.^52,53^ Applying the combination of using trauma databases for AI/ML/DL development, internal/external validation and the testing of the models should lead to the creation of a general medical AI/ML/DL algorithm. A custom diagram detailing a template for the creation of future trauma AI/ML/DL algorithms can be found in Figure 7.

In terms of progression from the results of this systematic review and meta-analysis, the next steps for future research should be a comparison of individual AI, ML and DL models. This review categorised all models together to enable a better comparison against conventional triage tools. However, it would be important to assess if either AI, ML or DL offer greater predictive ability of outcomes in trauma triage. There are already a wide array of models available such as gradient boosting (XGBoost), a ML algorithm where numerous weak learning classifiers are trained to combine together whilst learning from the results of previous combinations to produce better results or random forest, where learning is gained sequentially and is based on the performance of the previous stages. Different AI/ML/DL use different methods to achieve their specified outcomes. Therefore, it will be of high value to contrast the different methods and analyse for the most effective method.

Methodological deficiencies in this systematic review are primarily due to most studies being retrospective. A result of this means the data collected was not originally meant for research purposes, some databases in studies had missing data which may predispose the studies to confounding bias. However, this was mitigated by these studies through the exclusion of participants with missing data from the AI/ML/DL model development. In addition, the differences between each study created large heterogeneity with the results of our meta-analyses for all three outcomes. This was alleviated by using a random effects model in the meta-analyses. A common limitation of retrospective studies is the requirement of large sample sizes for rare events to be effective. This was easily managed in the studies due to the computing power of AI/ML/DL models which enables processing of large amounts of data.

Another limitation may have been in the effect measure of AUROC due to its deficiency in computing rare events with imbalanced data (where the number of negatives outweighs the positives) such as in-hospital mortality and critical care admission.^39^ When using AUROC, the false-positive rate (false positive/total actual negatives) does not dramatically decrease when the total negatives are large.^39,54^ A more suitable effect measure for imbalanced data would be the area under the precision-recall curve (AUPRC) as it considers the fraction of true positives in positive predictions therefore making it a more precise measure.^25,54^ However, AUPRC values and graphs are harder to interpret and do not consider true negatives at all, an important consideration for AI research, therefore making it a less popular option for AI/ML/DL researchers.

Thirdly, the differences in variables used in the development of various AI/ML/DL models were another limitation from this review. Whilst key variables such as age, sex and primary complaint were constant in all models, some studies included other variables to contribute to the learning of their AI/ML/DL models. This makes it difficult to ascertain the effect of the different variables on the predictive ability of AI/ML/DL models and how this ability could also change depending on the outcome being tested. Future research should be undertaken to evaluate models developed using different variables and whether this leads to better AI/ML/DL predictive ability for trauma outcomes, in addition to identifying the variables with the greatest impact on predictive ability.

## Conclusions

This systematic review and meta-analysis shows that AI/ML/DL models display greater accuracy at predicting key outcomes of mortality, hospitalisation and critical care admission compared to most conventional trauma triage tools. This is still an emerging and improving area of medicine which requires greater research, specifically in the form of prospective studies and randomised controlled trials. In order to benefit clinical policy and improve patient care, aims for future research on the use of AI/ML/DL models in trauma triage should be tailored to evaluating the clinical and economic effects and the potential creation of guidelines for the use of AI/ML/DL in trauma medicine.

## Supplemental Material

sj-docx-1-dhj-10.1177_20552076231205736 - Supplemental material for Exploring the effectiveness of artificial intelligence, machine learning and deep learning in trauma triage: A systematic review and meta-analysis Click here for additional data file. Supplemental material, sj-docx-1-dhj-10.1177_20552076231205736 for Exploring the effectiveness of artificial intelligence, machine learning and deep learning in trauma triage: A systematic review and meta-analysis by Oluwasemilore Adebayo, Zunira Areeba Bhuiyan and Zubair Ahmed in DIGITAL HEALTH
